# A Tale of Two Mice: Altered TREM2 Signalling Contributes to the Disparate Alzheimer's Disease Pathological Phenotypes Observed in ABI3‐Deficient Transgenic CRND8 and 5XFAD mice

**DOI:** 10.1002/alz70855_103298

**Published:** 2025-12-23

**Authors:** Deniz Ghaffari, Jennifer K Griffin, Ye Zhou, Fusheng Chen, Beatrice Acheson, Peter St George‐Hyslop

**Affiliations:** ^1^ University of Toronto, Toronto, ON, Canada; ^2^ Columbia University, New York, NY, USA

## Abstract

**Background:**

Alzheimer's disease (AD) remains the leading cause of dementia worldwide and currently lacks effective therapies. Recent studies have identified AD‐associated genetic variants in several microglial‐enriched genes, highlighting microglia as key contributors to disease pathogenesis and as promising therapeutic targets. These include several risk variants in ***Trem2*** and a rare coding risk variant in ***Abi3*** (rs616338:p. Ser209Phe). The impact of ABI3 deletion on AD pathology remains inconclusive as recent studies in ABI3‐deficient AD mouse models have produced conflicting observations. Specifically, the deletion of ABI3 ***reduces*** amyloid phenotypes in the TgCRND8 transgenic mouse model but ***exacerbates*** these phenotypes in the 5XFAD mouse model. We hypothesize that TREM2 signalling differences in C57 (genetic background of TgCRND8) and SJL (genetic background of 5XFAD) microglia contribute to this discrepancy.

**Method:**

Using primary mouse microglia derived from C57 and SJL mice, we analyzed TREM2 cleavage by ELISA and TREM2 signalling by stimulating the cells using an activating antibody and measuring SYK and PLCG2 phosphorylations (validated components of TREM2 signalling pathway) by western blotting. In addition, we compared TREM2‐dependent phagocytosis of amyloid‐beta by C57 and SJL microglia using IncuCyte S3 live imaging.

**Result:**

We observed significant alterations in TREM2 signalling in SJL microglia compared to C57 microglia. TREM2 cleavage was significantly lower in SJL microglia and following stimulation by an anti‐TREM2 activating antibody, SJL microglia demonstrated attenuated SYK and PLCG2 phosphorylations and reduced amyloid‐beta phagocytosis compared to C57 microglia.

**Conclusion:**

We have demonstrated that TREM2 signalling and the related AD‐associated functions are significantly altered in SJL microglia. In addition, previous co‐expression network analyses have suggested a close functional relationship between TREM2 and ABI3. A combined presence of altered TREM2 signalling and ABI3 deletion may explain the diverging phenotypes observed in TgCRND8 and 5XFAD mouse models. We are currently investigating other potential contributing factors such as differences in genetic background and AD transgenes expressed in these mouse models. We aim to uncover the molecular machinery that links TREM2 to ABI3 in microglia and to understand the impact of ABI3 S209F mutation on TREM2 signalling in microglia and in AD pathology, pathing the way towards identifying novel therapeutics.

